# Exploring the Mitochondrial Genomes of *Phoebe* Species (Lauraceae): Structural Dynamics and Functional Conservation

**DOI:** 10.1002/ece3.74281

**Published:** 2026-09-04

**Authors:** Ting Zou, Rong‐Rong Yan, Rong‐Hui Jiang, Hai‐Jun Ma, Yun‐Li Jiang, Guo‐Xiong Hu

**Affiliations:** ^1^ College of Life Sciences Guizhou University Guiyang China; ^2^ Key Laboratory of Plant Resource Conservation and Germplasm Innovation in Mountainous Region (Ministry of Education) Guizhou University Guiyang China; ^3^ Guizhou Academy of Forestry Guiyang China; ^4^ Institute of Botany Jiangsu Province and Chinese Academy of Sciences (Nanjing Botanical Garden Mem. Sun Yat‐Sen) Nanjing China

**Keywords:** comparative genomics, mitochondrial genome, mitogenome, nanmu, *Phoebe*, phylogenetic analysis

## Abstract

Plant mitochondrial genomes (mitogenomes) vary markedly in size and architecture despite generally slow rates of sequence evolution. *Phoebe* is an ecologically and economically valuable genus of Lauraceae, yet its mitogenome diversity remains poorly characterized. In this study, we newly sequenced, assembled, and annotated the mitogenomes of three nationally protected Class II wild plants (*P. bournei*, *P. chekiangensis*, *P. zhennan*) from China and compared their mitogenomic characteristics. The three assemblies were resolved into representative circular configurations ranging from 808 to 864 kb, with similar GC contents and conserved protein‐coding capacity. Each mitogenome contained distinct 41 protein‐coding genes, 27–28 transfer RNAs, and three ribosomal RNAs. Synteny analysis revealed extensive changes in homologous‐block order and orientation despite substantial sequence homology among the three species. Abundant repeats occurred predominantly in noncoding regions, while plastid‐derived fragments documented historical intracellular DNA transfer. The three species exhibited similar codon usage and predicted RNA‐editing patterns, whereas low synonymous divergence limited inference from pairwise ratios. Phylogenetic analysis based on mitochondrial protein‐coding genes recovered *Phoebe* as a well‐supported monophyletic lineage. These results reveal substantial structural divergence accompanied by conserved nucleotide composition and coding capacity, providing valuable data for further understanding the evolutionary variation of plant mitogenomes of *Phoebe* and the Lauraceae.

## Graphical abstract

This study assembled and comparatively analyzed the mitochondrial genomes of *P. bournei*, *P. chekiangensis*, and *P. zhennan*, revealing structural variations and functional conservation among these Phoebe species, thereby providing new data resources for understanding the evolutionary dynamics of plant mitochondrial genomes.

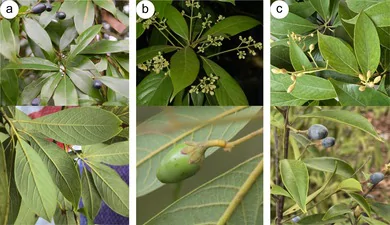

## 1 Introduction

Mitochondria are essential bioenergetic organelles responsible for cellular respiration and energy metabolism in eukaryotic cells (Chen et al. 2017; Christensen 2018). Following their endosymbiotic origin from an ancestral α‐proteobacterium, mitochondria have retained their own genome while undergoing extensive genome reduction and functional integration with the nuclear genome (Lu et al. 2024; Wang et al. 2024). Plant mitochondrial genomes (mitogenomes) display extensive divergence in genome size, structural architecture and intergenic regions, features that distinguish them from fungal and animal mitogenomes, both of which have been widely used for phylogenetic reconstruction (Cheng et al. 2025; Huang, Zhou, et al. 2026; Liu et al. 2025; Nethavhani et al. 2025). The size variation among plant mitogenomes assembled to date exceeds 200‐fold, spanning from 66 kb in *Viscum scurruloideum* Barlow (1996) (Skippington et al. 2015) to 18.99 Mb in *Cathaya argyrophylla* Chun & Kuang (1962) (Huang, Xu, et al. 2025), despite their relatively conserved coding sequences (typically 24 core genes and 17 variable genes). Although commonly represented as circular master molecules, plant mitogenomes may occur as dynamic populations of circular, linear, branched, multipartite, and recombination‐derived molecules (Chen, Yan, et al. 2025; Kozik et al. 2019; Wang et al. 2024; Wu et al. 2022). Such diversity in size and architecture is largely attributed to the expansion of non‐coding regions, primarily composed of repetitive elements, intracellular transfers, foreign insertions, and sequences of unknown origin (Donnelly et al. 2017; Hong et al. 2021; Li, Tang, et al. 2023). In contrast to this structural dynamism, plant mitochondrial coding sequences generally evolve slowly, with synonymous substitution rates lower than those of plastid and nuclear genomes (Drouin et al. 2008; Li, Wu, et al. 2025; Wolfe et al. 1987). Investigating these contrasting patterns is important for understanding plant mitogenome stability, structural evolution, and phylogenetic utility (Fan et al. 2025; Ma et al. 2022; Smith 2015). However, mitogenomes in angiosperms remain less extensively characterized than plastid genomes (plastomes) because of their structural complexity and associated assembly challenges (Wang et al. 2024). Recently, advances in high‐throughput sequencing technologies and improved assembly tools have begun to enable more comprehensive studies of plant mitogenomes (Bi et al. 2024; Han et al. 2025; He et al. 2023).

The genus *Phoebe* Nees (1831) belongs to the Lauraceae, comprising approximately 100 species distributed throughout tropical and subtropical Asia (Chen, Zeng, et al. 2020; Shao, Yang, et al. 2025; Wang et al. 2026). In China, around 30 species have been documented, mainly found in the Yangtze River basin and surrounding southern regions (Shi et al. 2023). Most *Phoebe* species are tall trees whose durable, decay‐resistant, aromatic, and attractively grained wood has long been valued for construction, furniture, and carving (Chen et al. 2023; Ding et al. 2015; He et al. 2017; Li et al. 2017). Beyond their economic importance, *Phoebe* species play vital ecological roles in subtropical forest ecosystems. This genus predominantly occurs in subtropical evergreen broad‐leaved forests, a characteristic and ecologically important vegetation formation of East Asia (Li, Liu, Song, et al. 2025; Meng et al. 2025). As large, long‐lived trees, they possess substantial carbon sequestration capacity with notable biomass carbon storage documented in plantation forests, and their extensive, deep‐reaching root systems provide a natural barrier against soil erosion (Hong et al. 2019; Wang 2011; Zhao et al. 2025). The conservation of such ecologically significant tree species is of critical importance, especially given that endangered tree species in tropical and subtropical Asia face distinct and often heightened vulnerabilities to environmental change (Hu et al. 2025). Four species of the genus *Phoebe*, including *Phoebe bournei* (Hemsl.) Y. C. Yang (1945), *P. chekiangensis* C. B. Shang (1974), *P. zhennan* S. K. Lee & F. N. Wei (1979), and *P. hui* W. C. Cheng ex Yen C. Yang (1945), are designated as Class II national key protected wild plants in China, all of which yield high‐value timber famous as golden‐thread nanmu owing to the unique shimmering golden luster of their heartwood (Shao, Xie, et al. 2025). Due to their significant economic and ecological value, these species have long been focal points of research and conservation, underscoring the importance of further genomic study.

To date, the plastomes of *Phoebe* species have been extensively investigated, consistently showing high conservation in genome size, structure, and gene content (Chen, Zeng, et al. 2020; Li et al. 2017; Liu et al. 2019). Chromosome‐scale nuclear genome assemblies are also available for *P. bournei* and *P. zhennan*, providing insights into their genomic characteristics and evolutionary histories (Chen, Sun, et al. 2020; Mao et al. 2025; Zou et al. 2026). To date, the mitogenome of *P. chekiangensis* has been publicly released (Tang et al. 2026), and two additional mitogenome sequences currently annotated as *P. bournei* (OR168698.1 and OR168699.1) are available in NCBI (Zhao et al. 2026). However, these *P. bournei* sequences were originally deposited as *Machilus pauhoi* Kaneh. (1930), resulting in inconsistent taxonomic labeling (Zhao et al. 2024). Consequently, well‐validated mitogenome resources and detailed comparative analyses of mitogenome variation among closely related *Phoebe* species are lacking. Here, we sequenced and assembled the mitogenomes of *P. bournei*, *P. chekiangensis*, and *P. zhennan* and performed a comparative analysis to (1) characterize their configurations, gene content, and compositional features, (2) examine repetitive sequences, mitochondrial plastid‐derived DNA fragments, and patterns of genomic rearrangement, (3) characterize codon usage, predicted RNA editing, and patterns of nonsynonymous and synonymous nucleotide substitutions, and (4) infer the phylogenetic relationships of these species based on mitochondrial data.

## 2 Materials and Methods

### 2.1 Plant Materials, DNA Extraction and Genome Sequencing

**FIGURE 1:**
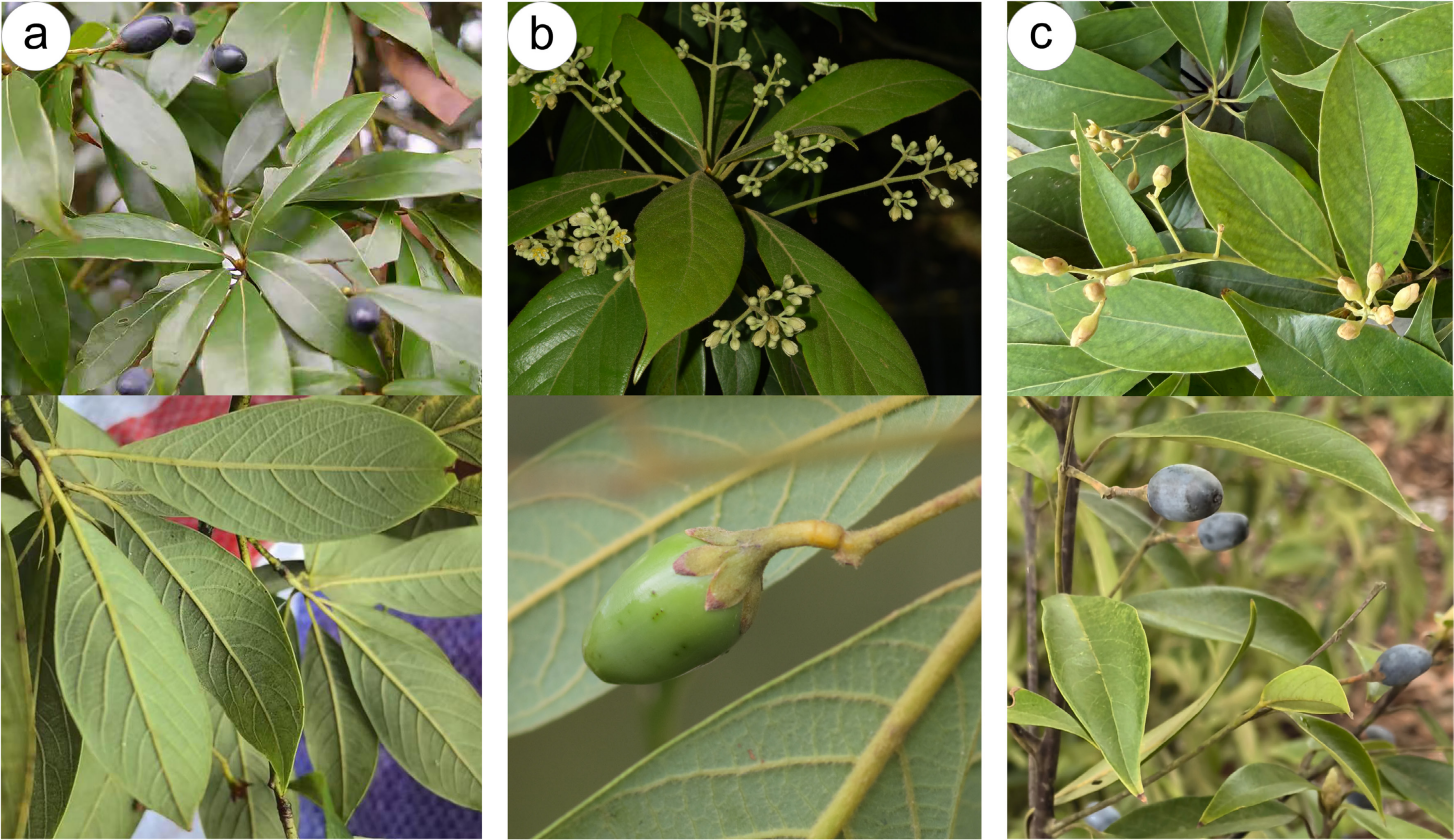
Morphological characteristics of three *Phoebe* species. (a) *P. bournei*; (b) *P. chekiangensis*; (c) *P. zhennan*.

Fresh leaves of *P. zhennan* and *P. bournei* were collected from the Guizhou Academy of Forestry, Guiyang, China, whereas samples of *P. chekiangensis* were obtained from Nanjing Botanical Garden Mem. Sun Yat‐Sen, Nanjing, China, in early 2025 (Figure 1). Species identity of all specimens was confirmed by Professor Yunli Jiang, a specialist in *Phoebe* taxonomy. Approximately 100 mg of fresh leaf tissue from each sampled individual was used for genomic DNA extraction. Total genomic DNA was extracted using a modified cetyltrimethylammonium bromide (CTAB) method (Doyle and Doyle 1987), and its purity and concentration were assessed using a NanoDrop spectrophotometer and a Qubit fluorometer, respectively. Approximately 5 μg of high‐quality genomic DNA from each sample was used for library preparation. PacBio HiFi libraries were constructed for *P. bournei* and *P. chekiangensis* and sequenced on the PacBio Revio platform, yielding at least 5 Gb of HiFi reads per species. An Oxford Nanopore Technologies library was constructed for *P. zhennan* and sequenced on the PromethION platform, yielding at least 10 Gb of ONT reads.

### 2.2 Mitogenome Assembly and Annotation

PacBio HiFi reads from *P. bournei* and *P. chekiangensis* were used directly for mitogenome assembly. For *P. zhennan*, raw ONT reads were first error‐corrected using Canu v2.2 (Koren et al. 2017), and only the corrected reads were used for subsequent assembly. Mitogenomes were assembled using PMAT v2.1.5 in autoMito mode (Bi et al. 2024). The resulting assembly graph was visualized and manually inspected using Bandage v0.8.1 (Wick et al. 2015). Low‐support branches and non‐target contigs were removed based on graph connectivity, sequence coverage, and sequence similarity to known plant mitochondrial sequences. The assembly graphs of *P. bournei* and *P. zhennan* each contained a single closed circular path and required no manual restructuring. In contrast, the *P. chekiangensis* assembly graph exhibited a repeat‐mediated figure‐eight topology, in which two circular regions shared a repetitive segment (Figure S1). The graph was resolved at the shared repeat based on graph connectivity, sequence coverage, and long‐read support, and one supported path was circularized and retained as a representative genome configuration for downstream annotation and comparative analyses. The mitogenome was annotated using PMGA (Li, Ni, et al. 2025) and GeSeq (Tillich et al. 2017), with *Cinnamomum camphora* (L.) J. Presl (1825) (NC_086632) and *Cinnamomum chekiangense* Nakai (1939) (NC_082065) as the reference sequences. All gene annotations were manually checked and adjusted in Geneious v2025.2 (Kearse et al. 2012) by comparison with reference organelle genomes. Mitogenome maps were generated using OrganellarGenomeDRAW (OGDRAW) (Greiner et al. 2019).

### 2.3 Repeat Sequence Analysis

Simple sequence repeats (SSRs) were identified using CPStools v.3.0 (Huang et al. 2024), with the minimum repeat thresholds of 10, 5, 4, 3, 3, and 3 repeat units for mono‐, di‐, tri‐, tetra‐, penta‐, and hexa‐nucleotide SSRs, respectively. Tandem repeats longer than 6 bp were identified using Tandem Repeats Finder v4.09 (Benson 1999) with default parameters. Dispersed repeats, including forward, palindromic, reverse, and complementary repeats, were detected using REPuter (Kurtz et al. 2001), with a minimum repeat length of 30 bp, a Hamming distance of 3, and a maximum of 5000 reported repeats.

### 2.4 Codon Usage Bias and RNA Editing Analyses

Protein‐coding genes (PCGs) were extracted from the annotated mitogenome using PhyloSuite v.1.2.3 (Zhang et al. 2020). Overall GC content, GC content at the first, second, and third codon positions (GC1, GC2, and GC3), and relative synonymous codon usage (RSCU) were calculated using CodonW v1.4.4 (Peden 2000). The effective number of codons (ENC) and GC content at synonymous third codon positions (GC3s) were also calculated using CodonW, and their relationship was visualized using an ENC–GC3s plot. RSCU = 1 indicates no codon usage bias, RSCU > 1 denotes preferentially used codons, and RSCU < 1 represents non‐preferred codons. Furthermore, C‐to‐U RNA‐editing sites of the PCGs were predicted using Deepred‐Mt v0.1.0 (Edera et al. 2021), and only sites with a probability above 0.9 were retained.

### 2.5 Nonsynonymous and Synonymous Substitution Analysis

The 41 mitochondrial PCGs shared by the three *Phoebe* species were extracted using PhyloSuite v.1.2.3 (Zhang et al. 2020). For genes with multiple copies, a single representative sequence was retained. Each gene was aligned separately using MAFFT v7 (Katoh and Standley 2013), with the coding frame maintained throughout the alignment. Pairwise nonsynonymous substitution rates (Ka) and synonymous substitution rates (Ks) were estimated using the codeml program implemented in PAML v4.10.9 (Yang 2007). Ka/Ks ratios were used to describe the relative accumulation of nonsynonymous and synonymous substitutions and were conventionally interpreted as indicating positive selection, neutral evolution, or purifying selection when greater than, equal to, or less than 1, respectively. When Ks was zero, the Ka/Ks ratio was undefined and was recorded as not available (NA).

### 2.6 Plastid‐Derived Insertions and Synteny Analysis

The plastomes of *P. bournei* (NC_034926), *P. chekiangensis* (NC_034925), and *P. zhennan* (NC_036143) were downloaded from the NCBI database. Homologous DNA sequences between mitogenome and plastome were identified using BLAST+ v2.7.1 (Chen et al. 2015), with an *e*‐value cutoff of 1 × 10^−6^. Retained homologous regions were classified as mitochondrial plastid‐derived DNA fragments (MTPTs), and their gene content was determined according to the corresponding plastome annotations. Synteny among mitogenomes was examined across *Machilus yunnanensis* Lecomte (1913) (PP968710.1), the three *Phoebe* species, and *Neocinnamomum delavayi* (Lecomte) H. Liu (1932) (PQ443074.1). Pairwise BLAST searches were conducted sequentially between *M. yunnanensis* and *P. bournei*, *P. bournei* and *P. chekiangensis*, *P. chekiangensis* and *P. zhennan*, and *P. zhennan* and *N. delavayi*. Only homologous alignments of at least 500 bp were retained. Syntenic relationships were visualized using PyGenomeViz, with red and blue ribbons representing homologous blocks in the same and opposite orientations, respectively.

### 2.7 Phylogenetic Inference

To reconstruct the phylogeny of the three *Phoebe* species, mitogenome sequences of 15 additional species were downloaded from the NCBI database, comprising 13 other Laurales species as the ingroup and two Magnoliales species as the outgroup (Table S1). Together with the three newly assembled *Phoebe* mitogenomes, a total of 36 shared PCGs were extracted using PhyloSuite v.1.2.3 (Zhang et al. 2020) and aligned in MAFFT v7 (Katoh and Standley 2013) with the auto strategy. Each independently aligned gene was combined into a multi‐sequence matrix. A maximum‐likelihood (ML) tree was inferred using IQ‐TREE v2.4.0 under the GTR + G substitution model, with branch support assessed using 1000 bootstrap replicates (Minh et al. 2020). The resulting phylogenetic tree was visualized and edited using iTOL (Letunic and Bork 2019).

## 3 Results

### 3.1 Mitogenome Features

**FIGURE 2:**
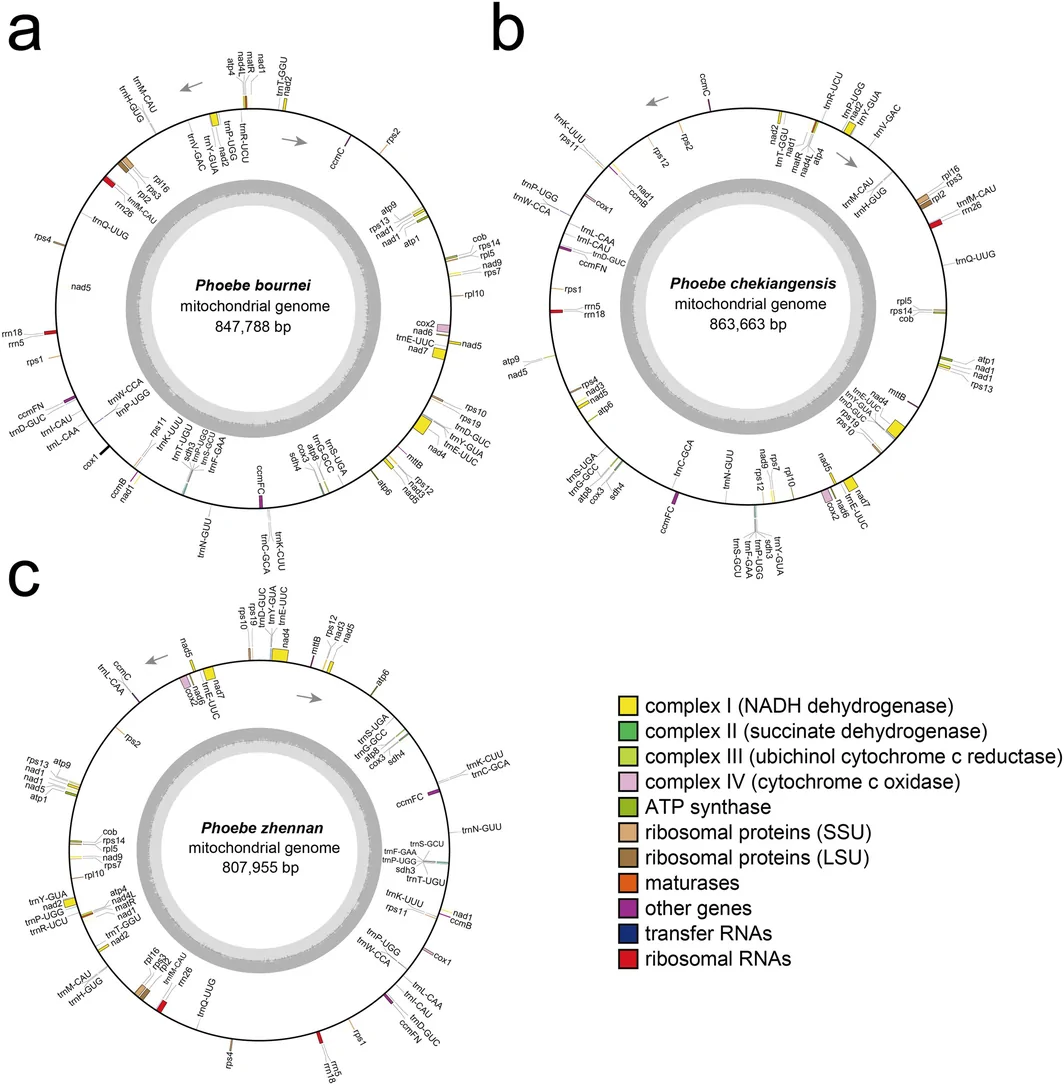
Circular maps of the representative mitogenome configurations of three *Phoebe* species. Genes shown inside and outside each circle are transcribed clockwise and counterclockwise, respectively. Colors indicate functional gene categories. Dark‐ and light‐gray regions in the inner circle represent GC and AT content, respectively. (a) *P. bournei*; (b) *P. chekiangensis*; (c) *P. zhennan*.

The three mitogenome assemblies were resolved into representative circular sequences of 847,788 bp in *P. bournei*, 863,663 bp in *P. chekiangensis*, and 807,955 bp in *P. zhennan* (Table 1, Figure 2). The total GC content was highly conserved across the three species, ranging from 47.04% to 47.11%. Each mitogenome contained 41 distinct PCGs, including the 24 genes conventionally classified as core mitochondrial genes (Table S2, Figure S2). *P. chekiangensis* contained an additional copy of rps12, resulting in a total of 42 PCG copies, whereas the remaining PCGs occurred as single copies in all three species. The mitogenomes also encoded 27–28 transfer RNAs (tRNAs) and three ribosomal RNAs (rRNAs). Variation in tRNA content was primarily associated with gene presence and copy number. Compared with *P. bournei*, *P. zhennan* had a duplicated *trnL‐CAA* and lacked the *trnV‐GAC*. *Phoebe chekiangensis* lacked the *trnT‐UGU* and *trnK‐CUU* but possessed three copies of the *trnY‐GUA*, while *P. bournei* and *P. zhennan* had only two copies (Table S2).

**TABLE 1:** Characteristics of the mitochondrial genome of three *Phoebe* species.

Species	Mitogenome size (bp)	GC (%)	PCGs	tRNAs	rRNAs	GenBank accession number
*P. bournei*	847,788	47.10%	41	28	3	PX826422
*P. chekiangensis*	863,663	47.11%	42	27	3	PX826423
*P. zhennan*	807,955	47.04%	41	28	3	PX826424

Abbreviations: PCGs, protein‐coding genes; rRNAs, ribosomal ribonucleic acids; tRNAs, transfer ribonucleic acids.

### 3.2 Repeat Sequence Analysis

**FIGURE 3:**
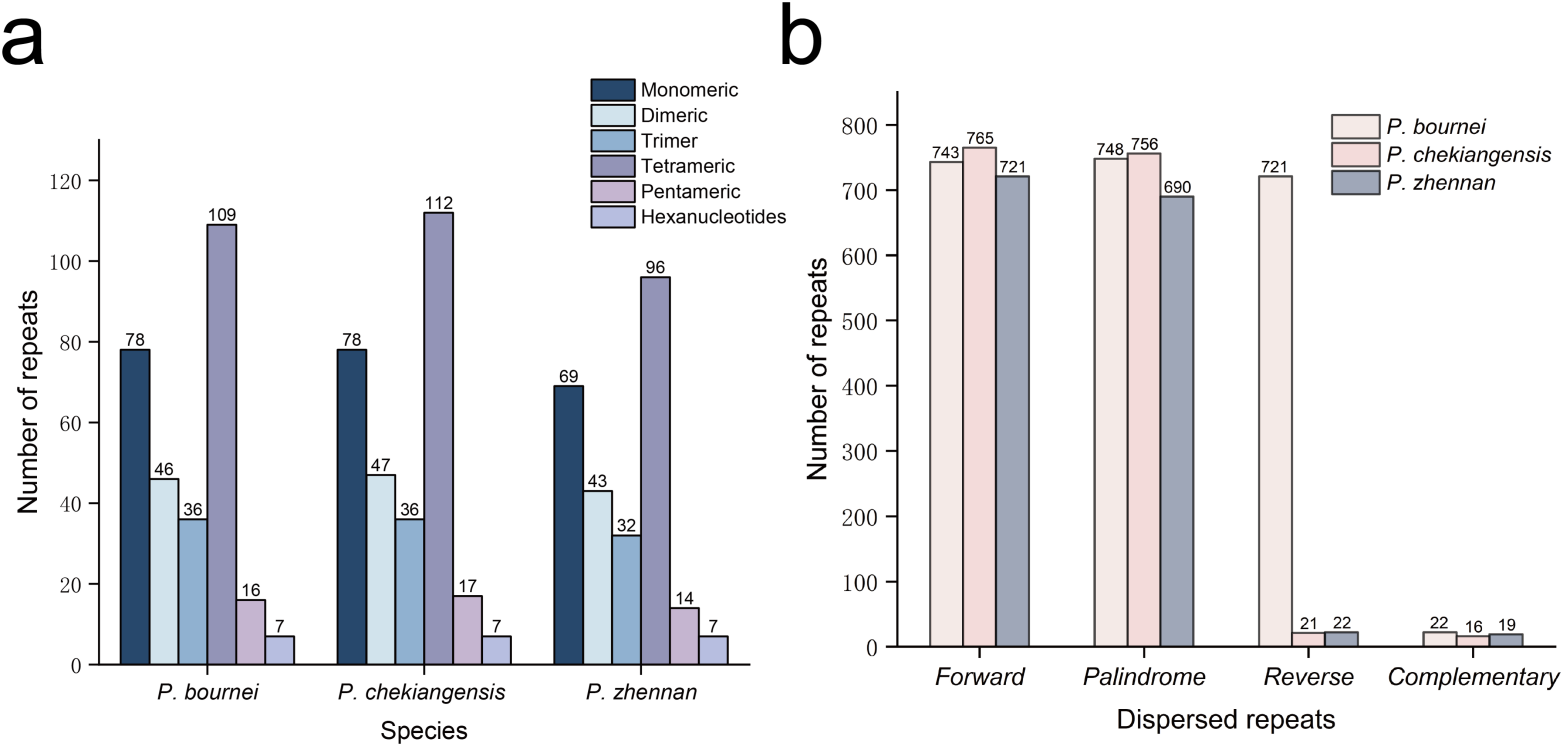
Repeat composition of the mitogenomes of three *Phoebe* species. (a) Numbers and types of simple sequence repeats (SSRs); (b) numbers and types of dispersed repeats.

A total of 850 SSRs were identified across the three *Phoebe* mitogenomes, including 292 in *P. bournei*, 297 in *P. chekiangensis*, and 261 in *P. zhennan* (Figure 3a, Table S3). All six SSR categories, from mono‐ to hexanucleotide repeats, were detected in each species. Tetranucleotide repeats were the most abundant, with 96–112 occurrences per species, followed by mononucleotide repeats with 69–78 occurrences. Hexanucleotide repeats were the least abundant, with seven occurrences in each mitogenome. Most SSRs were located in intergenic regions, with 251–287 occurrences per species, whereas only 4–5 SSRs occurred in genic regions and 4–5 in introns. In addition, 232 tandem repeats were identified, including 81 in *P. bournei*, 75 in *P. chekiangensis*, and 76 in *P. zhennan* (Table S4).

A total of 4545 dispersed repeats were detected, including 1535 in *P. bournei*, 1558 in *P. chekiangensis*, and 1452 in *P. zhennan* (Figure 3b, Table S5). Forward and palindromic repeats were predominant, with 721–765 and 690–756 occurrences per species, respectively. In contrast, reverse and complementary repeats were much less frequent, with only 16–22 occurrences of each type. Most dispersed repeats occurred in intergenic regions, with fewer located in genic regions and introns.

### 3.3 Codon Usage Patterns

**FIGURE 4:**
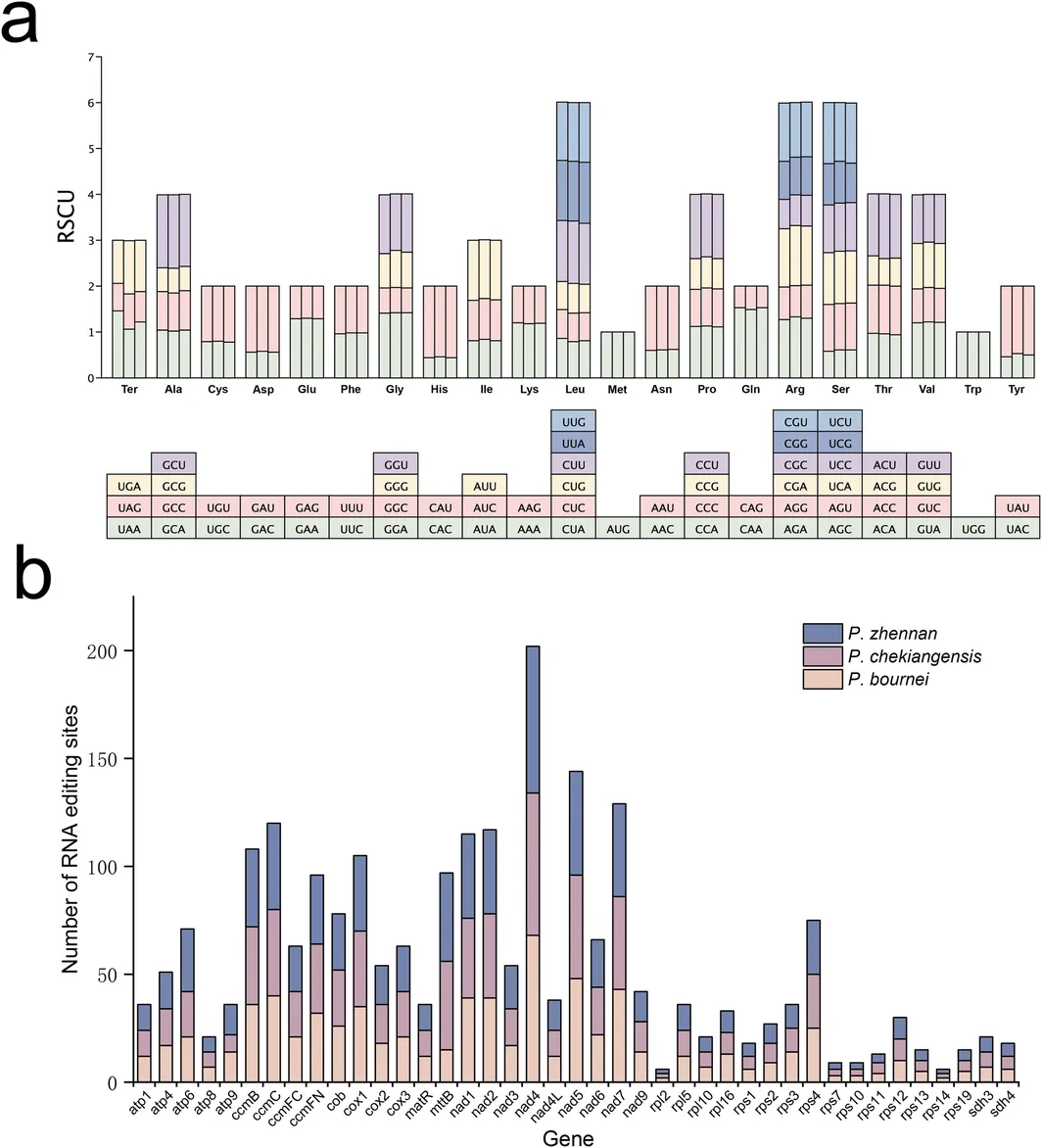
Codon usage and predicted RNA‐editing sites in the mitogenomes of three *Phoebe* species. (a) Relative synonymous codon usage (RSCU) of mitochondrial protein‐coding genes (PCGs), with the species ordered from left to right as *P. bournei*, *P. chekiangensis*, and *P. zhennan*. (b) Numbers of predicted C‐to‐U RNA‐editing sites in each PCG.

Codon usage analysis of the mitochondrial PCGs included 9953 codons in *P. bournei*, 8781 in *P. chekiangensis*, and 9220 in *P. zhennan* (Table S6). GC1, GC2, and GC3 ranged from 49.25% to 49.54%, 44.60% to 44.81%, and 39.02% to 39.48%, respectively, whereas the overall GC content of the coding sequences ranged from 44.41% to 44.54%. GC3 was consistently lower than GC1 and GC2, reflecting the preferential use of A‐ or U‐ending codons. ENC values ranged from 53.74 to 54.13, indicating relatively weak overall codon‐usage bias. RSCU profiles were highly similar among the three species (Figure 4a, Table S7). *Phoebe bournei* contained 31 codons with RSCU values greater than 1, whereas *P. chekiangensis* and *P. zhennan* each contained 32. Among the preferentially used codons, GCU for alanine (Ala) and CAU for histidine (His) had consistently high RSCU values across all three species. Methionine (AUG) and tryptophan (UGG) showed no codon preference because each was encoded by a single codon and therefore had an RSCU value of 1. The remaining non‐preferred codons had RSCU values below 1 (Table S7).

### 3.4 Predicted RNA‐Editing Sites

A total of 764, 773, and 797 C‐to‐U RNA editing sites were predicted across the 41 mitochondrial PCGs of *P. bournei*, *P. chekiangensis*, and *P. zhennan*, respectively (Figure 4b, Table S8). The number of predicted RNA editing events varied among different genes. The *nad4* gene contained the highest number of editing sites (66–68), followed by *nad5* with 48 sites. In contrast, only two or three RNA editing events were detected in each of the *rpl2*, *rps7*, *rps10*, and *rps14* genes. Predicted RNA editing sites were concentrated at the second codon position, which contained 470–492 sites and accounted for 60.8%–61.7% of the total. The first codon position contained 247–262 sites, whereas the third position contained only 43–46 sites, accounting for 5.4%–6.0% of all predicted sites.

### 3.5 Nucleotide Substitution Patterns

Pairwise Ka/Ks ratios were estimated for the 41 mitochondrial PCGs shared by the three *Phoebe* species (Table S9). Because synonymous substitutions were absent in many gene pairs, Ka/Ks ratios could be calculated for only 10 genes in the *P. bournei*‐*P. chekiangensis* comparison, six in the *P. bourne*i‐*P. zhennan* comparison, and nine in the *P. zhennan*‐*P. chekiangensis* comparison. The remaining 31–35 genes per comparison had Ks values of zero and were therefore recorded as NA. Among the calculable estimates, five genes had Ka/Ks ratios below 1 and five had ratios above 1 between *P. bournei* and *P. chekiangensis*. Between *P. bournei* and *P. zhennan*, five of the six calculable ratios were below 1, while *atp6* had a ratio of 1.1346. Between *P. zhennan* and *P. chekiangensis*, three genes had ratios below 1 and six had ratios above 1. The highest ratio was observed for nad2 (1.5522), followed by mttB (1.3395) and atp6 (1.1346). atp6 had a ratio above 1 in both comparisons involving *P. zhennan* but a ratio of zero between *P. bournei* and *P. chekiangensis*. Conversely, *mttB* and *rps10* had ratios above 1 in both comparisons involving *P. chekiangensis* but ratios of zero between *P. bournei* and *P. zhennan*.

### 3.6 Mitogenome Synteny and Structural Rearrangements

**FIGURE 5:**
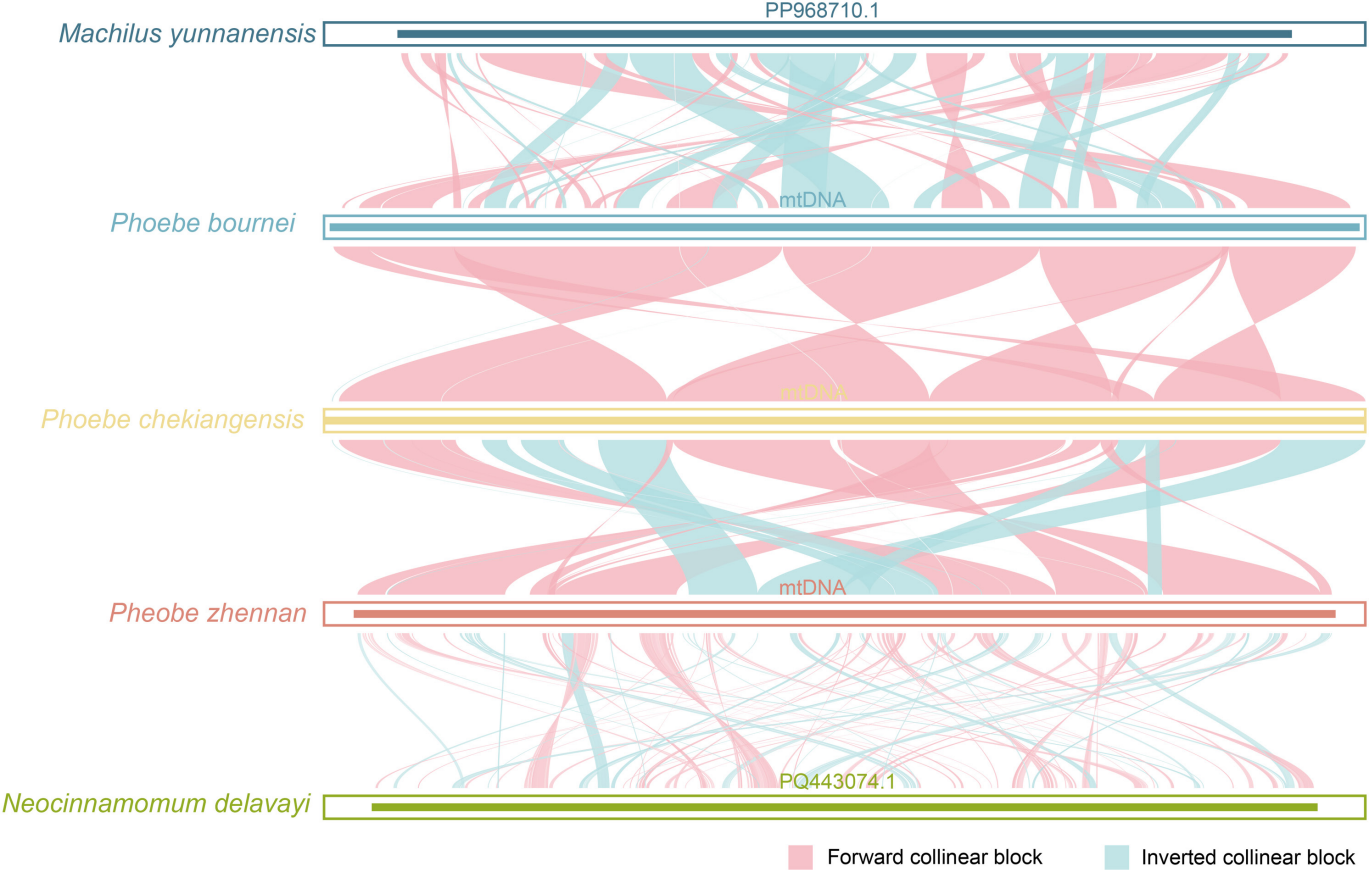
Pairwise mitogenome synteny among three *Phoebe* species and closely related taxa. Each horizontal bar represents a mitogenome sequence. Red ribbons connect homologous blocks in the same relative orientation, whereas blue ribbons connect homologous blocks in opposite relative orientations.

Pairwise synteny analysis among the three *Phoebe* species and their close relatives revealed extensive homologous regions but substantial differences in block order, orientation, and continuity (Figure 5, Table S10). Across the four pairwise comparisons, the number of syntenic blocks ranged from 11 to 172. The comparison between *P. bournei* and *P. chekiangensis* contained 11 blocks with a cumulative length of 847,196 bp, corresponding to 99.93% of the *P. bournei* mitogenome. The *M. yunnanensis* –*P. bournei*, *P. chekiangensis*–*P. zhennan*, and *P. zhennan*–*N. delavayi* comparisons contained 51, 24, and 172 blocks, respectively. Overall, although substantial sequence homology was retained among the examined mitogenomes, the arrangement and continuity of homologous regions varied markedly.

### 3.7 Mitochondrial Plastid‐Derived DNA Fragments

A total of 67, 67, and 65 MTPTs were identified in *P. bournei*, *P. chekiangensis*, and *P. zhennan*, respectively (Table S11). Their cumulative lengths were 48,798 bp in *P. bournei*, 48,600 bp in *P. chekiangensis*, and 44,836 bp in *P. zhennan*, accounting for 5.76%, 5.63%, and 5.55% of the corresponding mitogenomes, respectively. These fragments represented 31.92%, 31.80%, and 29.34% of the respective plastomes. In each species, 29 MTPTs matched regions in the large single‐copy region of the plastome, whereas the remaining 36–38 matched regions in the inverted repeats. Notably, no MTPTs corresponding to the small single‐copy region were detected. Collectively, the MTPTs contained five complete plastid PCGs (*rps7*, *ndhB*, *ycf4*, *cemA*, and *petN*) and 15 complete plastid tRNA genes. The remaining fragments corresponded to partial gene sequences or intergenic regions (Table S11).

### 3.8 Phylogenetic Relationships

**FIGURE 6:**
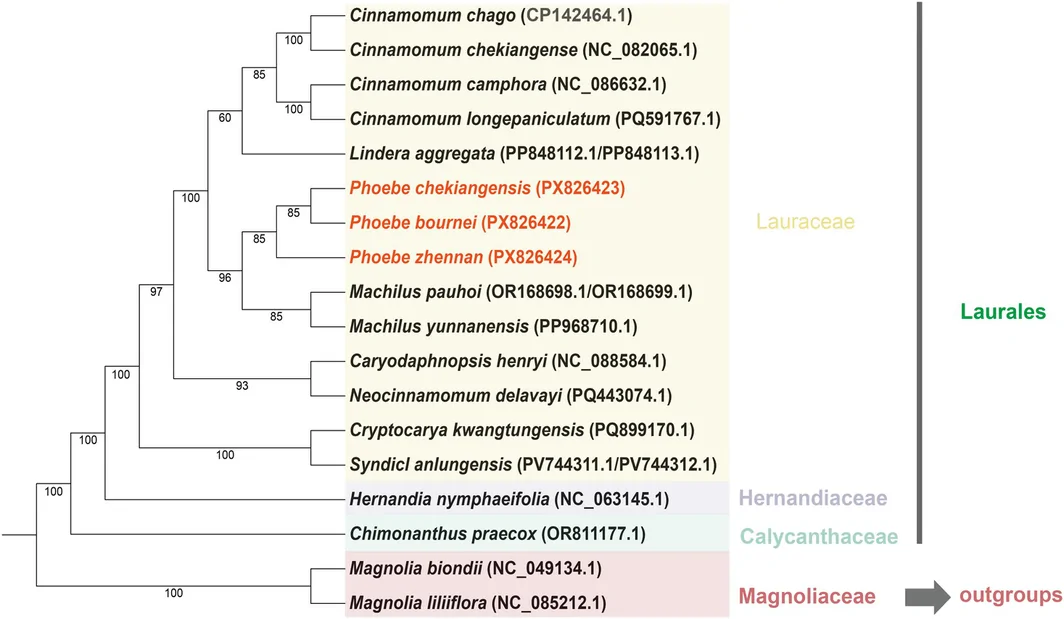
Maximum‐likelihood phylogeny inferred from 36 mitochondrial genes. Numbers on each node were bootstrap support values and the right side shows the family to which the species belongs.

Phylogenetic analyses based on the shared mitochondrial PCGs recovered Lauraceae as a well‐supported monophyletic clade (bootstrap support [BS] = 100; Figure 6). The three newly sequenced *Phoebe* species formed a monophyletic lineage (BS = 85) that was recovered as sister to *Machilus* Nees (1831). Within *Phoebe*, *P. bournei* and *P. chekiangensis* formed a sister pair (BS = 85), which together were sister to *P. zhennan*. Notably, the two accessions currently annotated as *P. bournei* in NCBI (OR168698.1 and OR168699.1), originally deposited under the name *Machilus pauhoi* and labeled as such in the present phylogenetic tree, did not cluster with the newly assembled *P. bournei* genome. Instead, they formed a strongly supported clade with *M. yunnanensis* , separating from the *Phoebe* lineage.

## 4 Discussion

### 4.1 Conservation and Structural Variation of *Phoebe* Mitogenomes

The three *Phoebe* mitogenomes were broadly similar in genome size, nucleotide composition, and gene content but differed in assembly‐graph topology. The single closed paths recovered for *P. bournei* and *P. zhennan* and the repeat‐associated figure‐eight graph of *P. chekiangensis* illustrate the structural complexity of plant mitogenomes. In particular, the representative circular configuration retained for *P. chekiangensis* should not be interpreted as its only possible in vivo structure, as plant mitogenomes are considered to occur as dynamic populations of alternative configurations generated through repeat‐mediated recombination (Fu et al. 2026; Kozik et al. 2019; Sloan 2013). Despite this structural variation, the similar GC contents and conserved set of 41 distinct PCGs indicate substantial conservation of nucleotide composition and protein‐coding capacity. This combination of variable genome architecture and conserved functional gene content is characteristic of many angiosperm mitogenomes (Adams et al. 2002; Wee et al. 2022).

Differences in tRNA content among the three species were associated primarily with gene presence and copy‐number variation. Some of these differences may be related to intracellular sequence transfer, as the MTPTs contained several intact plastid tRNA genes (Ma et al. 2015; Warren et al. 2025). However, sequence integrity alone does not demonstrate that the transferred genes are expressed or functional. Transcriptomic evidence would be required to determine whether they contribute to mitochondrial translation. The complete and partial plastid‐derived sequences identified here therefore provide evidence of historical intracellular DNA transfer rather than functional compensation. On the other hand, most repeats were located in noncoding regions, consistent with the extensive and variable intergenic regions characteristic of plant mitogenomes (Li, Han, et al. 2023; Qiu et al. 2025; Yan et al. 2026). Repeats can provide substrates for homologous recombination and thereby contribute to alternative genomic configurations and changes in sequence arrangement (Alverson et al. 2011; Kozik et al. 2019). Their abundance may therefore be associated with the structural variation observed among the three *Phoebe* mitogenomes, particularly the figure‐eight assembly graph of *P. chekiangensis*. Nevertheless, repeat abundance alone does not demonstrate active recombination. Confirmation would require the detection of alternative repeat‐flanking junctions and direct evaluation of their long‐read support.

### 4.2 Evolutionary Patterns of Mitochondrial Genes

RNA editing is a widespread post‐transcriptional regulatory mechanism in plant organelles and plays an essential role in correcting genomic information, modifying protein sequences, and maintaining organelle function (Sun et al. 2016; Edera et al. 2018). The similar numbers and distributions of predicted editing sites among the three *Phoebe* species suggest broadly conserved RNA‐editing patterns. The predominance of predicted editing sites at the second codon position, followed by the first position, is consistent with the general distribution of RNA editing in plant mitogenomes. Because editing at these positions frequently results in amino acid substitutions, this pattern suggests that most predicted editing events may affect mitochondrial protein sequences (Li et al. 2021; Yu et al. 2022). The three species also exhibited similar GC3, RSCU, and ENC patterns, including a general preference for A‐ or U‐ending codons and relatively weak overall codon‐usage bias. Comparable patterns have been reported in other plant mitogenomes (Li, Liu, Ma, and Liu 2025; Niu et al. 2022; Shi et al. 2024). Codon usage can be influenced by mutational composition, selection on translation, gene expression, and other evolutionary processes (Rocha 2004). Although the ENC–GC3s (Figure S3) distributions suggest that nucleotide composition alone may not fully explain the observed patterns, an ENC plot cannot independently distinguish the relative contributions of mutation and selection. The factors shaping mitochondrial codon usage in *Phoebe* therefore require further investigation using broader taxonomic and expression‐level data.

Pairwise Ka/Ks comparisons revealed heterogeneous substitution patterns among the mitochondrial PCGs. Several genes showed Ka/Ks ratios greater than 1 in at least one comparison, including *atp6*, *nad2*, *mttB*, and *rps10*. The *atp6* and *nad2* genes encode components of respiratory complexes V and I, respectively, and are directly involved in oxidative phosphorylation and cellular energy production. The *mttB* gene encodes a component of the mitochondrial twin‐arginine translocation pathway, whereas *rps10* encodes a small‐subunit ribosomal protein involved in mitochondrial translation (Carrie et al. 2016; Murcha et al. 2005). Elevated Ka/Ks ratios in mitochondrial genes associated with respiration and translation have also been reported in other plant lineages (Miao et al. 2024; Zhong et al. 2023; Zhu et al. 2025). These patterns suggest that the identified genes may have experienced lineage‐specific evolutionary pressures and could potentially contribute to functional differentiation in mitochondrial energy metabolism, protein translocation, and translation under differing environmental conditions. Nevertheless, Ks was zero for many genes, and only a limited number of Ka/Ks ratios could be calculated. These genes should therefore be regarded as candidates for adaptive evolution rather than as definitive evidence of positive selection. Broader taxon and population sampling, phylogenetically informed selection models, and functional analyses will be required to evaluate their potential roles in the environmental adaptation of *Phoebe* species.

### 4.3 Phylogenetic Implications and Collinearity

The phylogenetic tree inferred from mitochondrial PCGs recovered Lauraceae as a well‐supported monophyletic group, with *P. bournei*, *P. chekiangensis*, and *P. zhennan* forming a monophyletic lineage related to the genus *Machilus*. This result is congruent with previous phylogenetic reconstructions based on plastid and nuclear genomic data (Li et al. 2011; Li, Liu, Song, et al. 2025; Song et al. 2019). Plant mitochondrial genomes exhibit markedly lower nucleotide substitution rates than plastid and nuclear genomes (Wolfe et al. 1987), which limits their resolution of recent divergences. Large‐scale mitogenomic studies have demonstrated that mitochondrial PCG phylogenies are largely congruent with nuclear and plastome trees at the ordinal level and above (Hu et al. 2023; Lin et al. 2025; Xue et al. 2022), confirming their utility for resolving deep phylogenetic relationships. Within Lauraceae, mitochondrial genes have been shown to support relationships among major lineages, although establishing species‐level relationships based solely on mitochondrial data is not recommended (Song et al. 2024).

A noteworthy taxonomic inconsistency emerged in our phylogenetic analysis involving the two accessions OR168698.1 and OR168699.1. These accessions were originally identified as *Machilus pauhoi* in the preprint by Zhao et al. (2024), but were subsequently reassigned to *P. bournei* in the final publication (Zhao et al. 2026) and are currently annotated as such in the NCBI database. In our mitochondrial PCG phylogeny, both accessions clustered with *M. yunnanensis* in a strongly supported clade, rather than with the newly assembled *P. bournei* genome. This placement is consistent with their original assignment to *Machilus*. Several explanations could account for this discordance: sample misidentification, contamination or sequence‐labeling errors during data generation, assembly artifacts arising from short‐read sequencing, or genuine mito‐nuclear discordance due to historical introgression or incomplete lineage sorting. The three *Phoebe* species sequenced in this study were identified through rigorous morphological examination, providing high confidence in their taxonomic identities (Figure 1). Given that *Phoebe* and *Machilus* are well‐established as reciprocally monophyletic genera (Li et al. 2011; Li, Liu, Song, et al. 2025), and that our vouchered *P. bournei* occupies the expected position within the *Phoebe* clade, we consider misidentification of the source material the most parsimonious explanation and recommend re‐examination of the original voucher specimens.

The synteny results demonstrate that extensive changes in block order and orientation can occur despite substantial conservation of homologous sequences among *Phoebe* mitogenomes. This contrast supports a partial decoupling between the rapid evolution of mitochondrial genome architecture and the relative conservation of coding sequences. Abundant dispersed repeats may provide substrates for the recombination processes underlying such structural variation, although the present study did not directly associate individual rearrangement breakpoints with particular repeats (Cole et al. 2018; Sun et al. 2022). A similar combination of extensive structural rearrangement, rapid repeat turnover, and conserved phylogenetic signal has also been reported in Digitalideae (Mower et al. 2026).

## 5 Conclusion

This study provides a consistent comparative characterization of the mitogenomes of *P. bournei*, *P. chekiangensis*, and *P. zhennan*. The three mitogenomes exhibited similar nucleotide composition and conserved protein‐coding capacity but differed in assembly‐graph topology and homologous‐block arrangement, revealing substantial structural variation among closely related species. Repetitive sequences and MTPTs contributed to their noncoding sequence diversity, although their roles in recombination and mitochondrial function require further investigation. The broadly similar gene complements, codon‐usage profiles, and predicted RNA‐editing patterns indicate conservation of fundamental mitochondrial features across the three species. The mitochondrial phylogeny supported a close relationship among the three newly assembled *Phoebe* mitogenomes and between *Phoebe* and *Machilus*. Together, these findings expand mitogenomic resources for these nationally protected *Phoebe* species and provide valuable data for further studies of mitogenome evolution and taxonomic relationships within Lauraceae.

## Author Contributions

**Ting Zou:** formal analysis (lead), visualization (lead), writing – original draft (lead), writing – review and editing (equal). **Rong‐Rong Yan:** formal analysis (supporting), writing – review and editing (equal). **Rong‐Hui Jiang:** resources (equal), writing – review and editing (equal). **Hai‐Jun Ma:** resources (equal), writing – review and editing (equal). **Yun‐Li Jiang:** resources (lead), writing – review and editing (equal). **Guo‐Xiong Hu:** conceptualization (lead), writing – review and editing (equal).

## Funding

This research was funded by the Key Research Project of the Guizhou Forestry Bureau (QLKH [2026] ZD004); Investigation on *Phoebe* Resources in Chishui Alsophila National Nature Reserve (202641); Special Survey Project on Rare and Endangered Wild Plants in the Kuankuoshui National Nature Reserve (2025‐1); the Guizhou Provincial Science and Technology Plan Project (Qiankehe Zhongyindi [2023] 029); the 2025 Cultivation of *Phoebe bournei* Progeny Seedlings and Establishment of Regional Trial Plantations (2025‐ZM‐14); the Monitoring and Re‐assessment Survey Project for Ancient and Famous Trees in Guizhou Province; and the Innovative Talent Team Project of Seedling Breeding and Plantation Cultivation for Precious Tree Species in Guizhou (Qiankehe Platform Talents‐CXTD[2023]006).

## Conflicts of Interest

The authors declare no conflicts of interest.

## Supporting information


**Figure S1:** The structural landscape of *Phoebe* mitogenomes.
**Figure S2:** Comparison of mitochondrial gene presence in *Phoebe*.
**Figure S3:** ENC‐plot of PCGs.

**Table S1:** Mitochondrial genomic information of 18 species for the construction of phylogenetic trees.
**Table S2:** Gene features in the mitogenome of *Phoebe*.
**Table S3:** SSRs in the mitogenome of *Phoebe*.
**Table S4:** Tandem repeat sequences in the mitogenome of *Phoebe*.
**Table S5:** Number of long dispersed repeats in the mitogenome of *Phoebe*.
**Table S6:** GC content of different positions of codon in the mitogenome of *Phoebe*.
**Table S7:** Codon usage bias of mitochondrial PCGs of *Phoebe*.
**Table S8:** Predicted RNA editing sites in 41 PCGs.
**Table S9:** The Ka/Ks ratios of each gene in the mitogenome of *Phoebe*.
**Table S10:** Homologous colinear blocks of *Phoebe* with closely related species.
**Table S11:** Homologous fragments of mitogenome and plastome.

## Data Availability

The newly assembled and annotated mitogenomes of *P. bournei*, *P. chekiangensis*, and *P. zhennan* have been deposited in the NCBI repository (https://www.ncbi.nlm.nih.gov/), with accession numbers PX826422, PX826423, and PX826424, respectively.
