# Supplementary material for: Exploring the Mitochondrial Genomes of Phoebe Species (Lauraceae): Structural Dynamics and Functional Conservation
Source: Ecol Evol. 2026 Sep 4;16(9):e74281. doi: 10.1002/ece3.74281 (PMC13543305; doi:10.1002/ece3.74281)
Supplement: Supplementary file 1 [file ECE3-16-e74281-s002.docx]

**Figure S1** The structural landscape of *Phoebe* mitogenomes. The numbers indicate average read coverage (×) for each contig. Connections between contigs denote potential recombination or repeat-mediated linkage relationships inferred from assembly graph analysis.

**Figure S2** Comparison of mitochondrial gene presence in *Phoebe*

**Figure S3** ENC-plot of PCGs. a. *P. bournei*; b. *P. chekiangensis*; c. *P. zhennan*
